# Cavernous Sinus Thrombosis Secondary to *Streptococcus Constellatus Pharynges*

**DOI:** 10.1007/s12070-024-04511-3

**Published:** 2024-02-05

**Authors:** Natalia Jaramillo-Ángel, Miguel Saro-Buendía, Joan Carreres Polo, Raul Mellidez Acosta, Agustín Alamar Velázquez, Miguel Armengot Carceller

**Affiliations:** 1 Departamento de Otorrinolaringología, Hospital Universitario y Politécnico La Fe, València, España; 2 Departament de Cirugía, Facultat de Medicina i Odontología, Universitat de València, València, España; 3 Departamento de Radiología, Hospital Universitario y Politécnico La Fe, València, España

**Keywords:** *Streptococcus constellatus pharyngis*, Cavernous Sinus Thrombosis, Gram Positive Commensal Bacterium, Invasive Pyogenic Infections, Head and neck Abscesses, Brain Abscesses, Long term Antibiotherapy, Anticoagulation

## Abstract

*Streptococcus constellatus pharyngis* is a gram-positive commensal bacterium commonly found in the oropharynx, gastrointestinal and urogenital tracts. It might be an aggressive opportunistic pathogen causing invasive pyogenic infections in sterile areas, mostly as peritonsillar and orofacial abscesses. We report the case of a 6-year-old girl, who presented multiple head and neck abscesses and bilateral cavernous sinus thrombosis secondary to *Streptococcus constellatus pharyngis*. Cavernous sinus thrombosis, consequent to this microorganism, has not been reported to date in the literature. Due to the invasive features of this pathogen, a long-term antibiotherapy (up to 9 months) is required. Additionally, a surgical drainage is indicated in case of head and neck, or brain abscesses, larger than 20 or 25 mm respectively. Anticoagulation should be considered in case of venous thrombosis. The interest of this case is not only based on the rarity and severity of the disease, but also on the success of medical and surgical therapy (including long- term antibiotics, anticoagulation and two surgical procedures). This experience may serve as a guide to treat future cases.

**Supplementary Information:**

The online version contains supplementary material available at 10.1007/s12070-024-04511-3.

## Introduction

*Streptococcus constellatus pharyngis* (SCP) is a gram- positive bacteria, usually colonizing the upper respiratory, digestive, and reproductive tracts. As a member of the group *Streptococcus milleri*, SCP might behave as an opportunistic pathogen causing invasive pyogenic infections in sterile locations (usually in the head and neck region) [1, 2]. There are reports of peritonsillar and orofacial abscesses caused by SCP [3, 4], however, we did not find cases of cavernous sinus thrombosis (CST) secondary to SCP infections [5]. CST is a potentially fatal complication of orofacial infections. This condition is very rare (0.2 to 1.6 cases per 100,000 persons, per year) and patients are usually children and newborns. Its clinical presentation includes fever, headache, periorbital edema and/or ophthalmoplegia [6]. 

Our aim is to report a case of severe and multifocal head and neck infection caused by SCP. Moreover, this is the first report of CST secondary to a head and neck SCP infection.

## Clinical Case

A 6-year-old girl, with no significant medical history and complete vaccination, presented with a 72-hour history of fever, odynophagia, left otalgia and drowsiness. In the last 12 h, she developed a right maxillary and periorbital swelling. Lab tests showed a neutrophilic leukocytosis and increased levels of C-reactive protein (439 mg/L) and procalcitonin (99 ng/ml). Computed tomography (CT) showed a non-complicated pansinusitis, leading to her admission in a pediatric hospitalization ward at another center. Therapy consisted of anti-inflammatory drugs and antibiotics (meropenem 40 mg/kg/day and vancomycin 60 mg/kg/day).

**Fig. 1:**
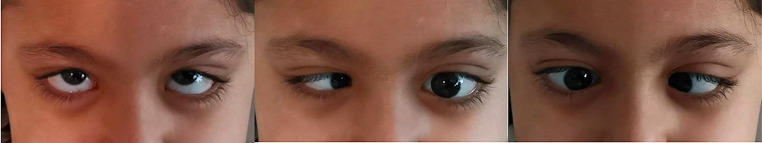
Esotropia resulting from impaired ocular abduction, a characteristic manifestation of 6th cranial nerve paralysis

**Fig. 2:**
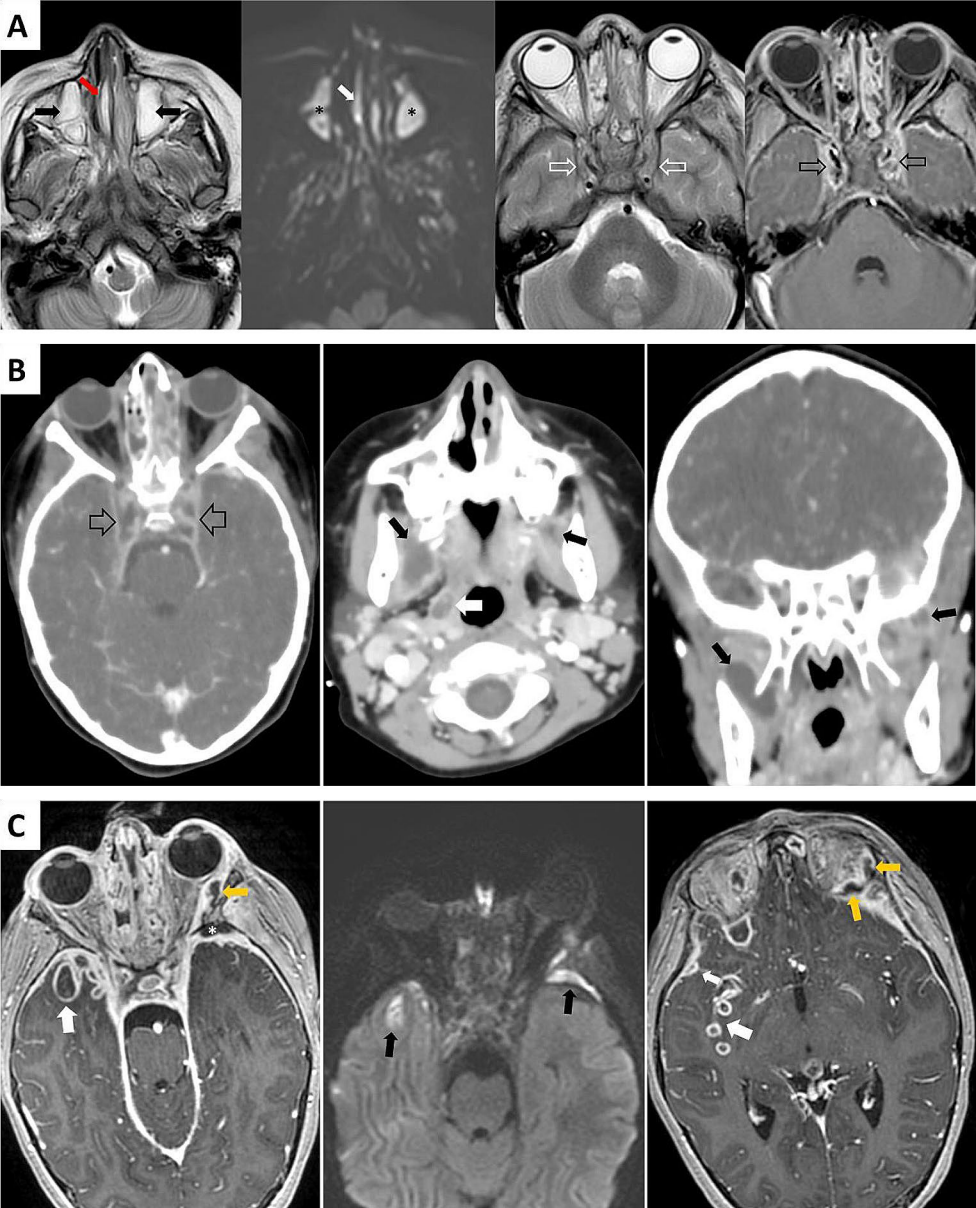
**A**: MR after bilateral VI cranial nerve paralysis debut. MRI T2 sequence revealed a sinusitis- pattern with pus occupying both maxillary sinuses (black arrows), showing restricted diffusion (asterisks). A right para septal abscess was observed (red arrow) showing restricted diffusion (white arrow). Bilateral cavernous sinus thrombosis was detected, it is characterized by enlargement and occupation of both cavernous sinuses on T2 sequence (white hollow arrows) and filling defects on T1 3D post-contrast sequence (black hollow arrows). **B**: CT 5 days after the first surgical procedure. Bilateral worsening of the cavernous sinus thrombosis. Large bilateral filling defects are observed (hollow black arrows). Both masticatory spaces are affected with abscesses in the right pterygoid muscle and the left temporal muscle insertion (black arrows). Also, a right peritonsillar abscess was present (white arrow). **C**: MR 1 week after the second surgical procedure. It demonstrated a left orbital abscess in the post-contrast T1 sequence (orange arrows). Intraparenchymal abscesses were observed in the right temporal lobe, also in the right insula (white arrows). Anepidural empyema contacted the left greater wing of the sphenoid bone (asterisk). Overall, the abscesses showed a restricted diffusion (black arrows) due to the presence of pus

In the next days, she presented an inability to abduct both eyes (Fig. 1). A sixth cranial nerve palsy secondary to CST was suspected. In this scenario, ceftriaxone (40 mg/kg/day) was added to meropenem and vancomycin. Magnetic resonance (MR) demonstrated bilateral maxillary sinusitis, a right paraseptal abscess and bilateral CST (Fig. 2A). Subsequently, she was referred to our center.

Upon arrival antimicrobial therapy was modified to cefotaxime (300 mg/kg/day) and metronidazole (30 mg/kg/day). Medical therapy also included anti- inflammatory drugs (methylprednisolone 1 mg/kg/day) and anticoagulation (enoxaparin). A surgical procedure (endoscopic endonasal approach) was performed to drain both maxillary sinuses, anterior ethmoidal cells, and the right paraseptal abscess. Samples of purulent fluid revealed the presence of SCP, prompting the addition of vancomycin to the treatment regimen.

In the fifth postoperative day, she presented a clinical worsening of the previous symptoms, an increment of the CST extension was seen on CT, with bilateral masticatory space abscesses and right peritonsillar abscess (Fig. 2B). A second surgical procedure (transoral and endoscopic endonasal approach) was performed to drain the abscesses. One-week later symptoms persisted, and a subsequent MR revealed a left orbital abscess, intraparenchymal temporal abscesses and an epidural empyema (Fig. 2C). At this point, neurosurgeons recommended a close follow-up avoiding surgery, due to the risk/benefit ratio of a third intervention. As a result, gentamycin was added to medical therapy, and methylprednisolone was gradually reduced.

In the next days, the patient showed an overall clinical- analytical improvement. One week later, a follow-up MR showed the resolution of the previous epidural empyema and left orbital and right temporal abscesses. Also, the bilateral CST seemed to be resolved. Given the overall improvement, the following week the patient was discharged with oral antibiotics (ceftriaxone 300 mg/kg/day and metronidazole 300 mg/kg/day) and subcutaneous enoxaparin.

Throughout the hospitalization and during outpatient visits, a study of immunoglobulins, cytometry panel, serology, and hemostasis was conducted, ruling out any underlying immune, hematological, or infectious disease.

After a month of domiciliary therapy, she showed a complete clinical recovery confirmed by an MR that revealed the absence of abscesses.

## Discussion

SCP might behave aggressively, causing invasive pyogenic infections in sterile locations. Along with *Streptococcus anginosus and intermedius* (EGA), SCP may cause pharyngitis, peritonsillar abscess, dental infection, Lemierre syndrome, sinusitis, otitis media, orbital abscess, and central nervous system infections [2]. While the precise mechanism of its virulence remains unclear, SCP polysaccharide capsule is thought to play a contributing role. The infection may spread by contiguity or hematogenous dissemination, resulting in metastatic abscesses (brain, liver, spleen, subdural space, bone, or heart) [1, 3]. Risk factors associated with the development of multiple abscesses, due to this pathogen, include underlying medical conditions such as cancer, diabetes, hypertension, and immunosuppression, also poor dietary habits, smoking, alcohol and drug consumption [4].

Long-term antibiotic therapy is required due to the invasive nature of SCP, the duration of therapy may be extended up to 9 months. Commonly used drugs are beta-lactams, lincosamides, cephalosporins, fluoroquinolones and metronidazole [1]. Surgical procedure should be considered in case of abscesses larger than 20 mm, airway compromise or septic contexts. However, a brain abscess drainage should be considered in case of mass effect or if is larger than 25 mm [6].

CST might be secondary to the hematogenous spread of microorganisms. This may occur due to retrograde flow from the emissary veins, facilitated by the absence of valves in the dural sinus. This mechanism allows the spread of thrombus into dural structures causing meningitis, dural empyema or cerebral abscess [5]. In CST, the facial and ophthalmic vein drainage is compromised. This results in ptosis, proptosis, chemosis, periorbital edema, pain, and a decreased visual acuity. Additionally, the cranial nerves within the cavernous sinus may be affected. Related symptoms would be V1-2 dermatome paresthesia, facial pain or diplopia due to ophthalmoplegia [5]. According to guidelines, therapy for CST shall include anticoagulants (unfractionated or low-molecular-weight heparin) and antibiotics (against Staphylococcus and anaerobes). Anticoagulation should be used for several weeks (even months) to reduce the rates of mortality (from 40 to 14%) and neurological morbidity (from 61 to 31%) [5].

## Conclusions

An SCP infection may lead to severe head and neck abscesses and intracranial complications (brain parenchymal abscesses and CST). A precise diagnostic work-up and a multidisciplinary therapeutic approach (including medical and surgical strategies) are needed to achieve optimal outcomes.

Our patient’s case serves as a notable illustration; despite her absence of comorbidities and the absence of other bacteria in cultures, she endured a prolonged course of illness, marked by the development of multiple craniofacial infection sites and CST. Her recovery involved a prolonged course of antibiotic and two surgical interventions to effectively control the infection.

### Electronic Supplementary Material

Below is the link to the electronic supplementary material.

Supplementary Material 1
