# Supplementary material for: Cavernous Sinus Thrombosis Secondary to Streptococcus Constellatus Pharynges
Source: Indian J Otolaryngol Head Neck Surg. 2024 Feb 5;76(3):2824–7. doi: 10.1007/s12070-024-04511-3 (PMC11169213; doi:10.1007/s12070-024-04511-3)
Supplement: Supplementary file 1 — Supplementary Material 1 [file 12070_2024_4511_MOESM1_ESM.pdf]

ANEXO 1
AUTORIZACION DE USO, CAPTACIÓN Y DIFUSIÓN DE IMÁGENES DE PACIENTES CON FINES DOCENTES Y DE INVESTIGACIÓN

DATOS DEL/ LA PROFESIONAL DEL DEPARTAMENT DE SALUT VALÈNCIA LA FE

Natalia Jaramillo Angel
Nombre y Apellidos del/ la Profesional Sanitario/a o Persona que solicita el uso de las imágenes

Perteneciente al Servicio de: Otorrinolaringología

Nº de Colegiado/a 464626833

Correo electrónico GVA: jaramillo_nat@gva.es

Solicita a través de este documento, autorización para grabar (explicación de la técnica/ intervención que se vaya a grabar) Fotografía, RM, TC

Con el objetivo de (Justificar y motivar la importancia y pertinencia de esta grabación) publicación, comunicaciones, investigación.

Promovida por la Empresa / Entidad:(*) Hospital Universitario y Politécnico La Fe
Que está previsto se difunda/utilice en Revistas científicas.

DATOS DEL / LA PACIENTE

Conocido esto, y de acuerdo a lo establecido en la Ley Orgánica 15/99 de Protección de Datos; el Reglamento General de Protección de Datos 2016/679; la Ley Orgánica 1/1982 de Protección Civil del Derecho al honor, a la intimidad personal y familiar y a la propia imagen; la Ley 41/2002 de Autonomía del Paciente y la Ley 10/2014, de Salud de la Comunidad Valenciana:

D/Dña Kawthar Laifa mayor de edad,
(Nombre y apellidos del paciente)

DNI________ Telf. 631888826 Domicilio calle Mare de deu de la Purificació 23 Torrent

Nº de Historia Clínica Paciente: 6972508 Nº de SIP 11471112

(*) Se debe completar en caso de que la toma y difusión de imágenes conlleve explotación comercial o esté promovida por alguna empresa externa.

Scanned with CamScanner

Se debe completar este apartado sólo en caso de tratarse de Paciente menor de Edad (MADRE- PADRE TUTOR/A LEGAL- Necesaria firma y datos de dos progenitores en caso de Menor de Edad)

D/Dña ______________________________ mayor de edad,
(PADRE / O TUTOR/A LEGAL)

DNI______________Telf.______________

D/Dña WAHIBA LAIFA mayor de edad,
(MADRE O TUTOR/A LEGAL)

DNI Y1923497K Telf. 631888826

1.- Manifiesto haber sido informado/a que durante mi proceso asistencial, o del menor al que represento, siempre que así lo autorice expresamente, y sin que mi decisión en un sentido o en otro afecte al trato dispensado por el personal sanitario, se podrán tomar fotografías o grabar imágenes que serán conservadas por el Servicio del Hospital La Fe que me solicita la grabación, a donde me podré dirigir para ejercitar los derechos que en materia de protección de datos me asisten.

2.- De igual modo, manifiesto haber sido informado/a que estas imágenes y datos clínicos se podrán transmitir y difundir con fines de Investigación y Docencia, preservando mi identidad o la de mi hijo/a menor de edad de forma confidencial, e incluso ser explotados por terceros.

3.- Que autorizo a que se tomen imágenes de mi persona, o del menor al que represento, (Marque lo que corresponda)

DE FORMA SÍ RECONOCIBLE[1] ☒
DE FORMA NO RECONOCIBLE ☐

4.- Que he sido informado/a que el Hospital La Fe no se hace responsable de cualquier otro uso indebido que se haga de estas imágenes y/o videos por parte de terceros, una vez éstas sean difundidas.

Y para que así conste a todos los efectos, firmo la presente autorización,

En València, a 19 de junio de 2023

Firmado______________________
(Paciente cuando sea mayor de 14 años)

____
Firmado Madre/ tutora legal (en caso de menor de edad)

____
Firmado Padre/ tutora legal (en caso de menor de edad)

[1] En caso de que el/la menor salga de forma reconocible se deberá notificar por parte del profesional implicado también a la Fiscalía del Menor

Scanned with CamScanner

REVOCACIÓN AUTORIZACIÓN

Mediante el presente documento declaro la revocación de la autorización firmada el día: ________________________en la que consentí la realización de la toma de imágenes y video para los fines arriba indicados, conociendo las limitaciones que este procedimiento de revocación conlleva.

Nombre ____

(Si corresponde) Representante legal de ____

Fecha:_________________________

Firma Paciente o Representante legal ____

València, a __ de ____________ de 20__

Scanned with CamScanner
